# Phenotypic and molecular marker analysis uncovers the genetic diversity of the grass *Stenotaphrum secundatum*

**DOI:** 10.1186/s12863-020-00892-w

**Published:** 2020-08-12

**Authors:** Ying Luo, Xiujie Zhang, Jiahong Xu, Yao Zheng, Shouqin Pu, Zhizhen Duan, Zhihao Li, Guodao Liu, Jinhui Chen, Zhiyong Wang

**Affiliations:** 1 Key Laboratory of Genetics and Germplasm Innovation of Tropical Special Forest Trees and Ornamental Plants, Ministry of Education/Engineering Research Center of Rare and Precious Tree Species in Hainan Province, College of Forestry, Hainan University, Haikou, 570228 People’s Republic of China; 2 Hainan Biological Key Laboratory for Germplasm Resources of Tropical Special Ornamental Plants, College of Forestry, Hainan University, Haikou, 570228 People’s Republic of China; 3 Chinese Academy of Tropical Agricultural Science, Haikou, 570228 People’s Republic of China

**Keywords:** *Stenotaphrum secundatum*, Phenotype, Molecular markers, Single-nucleotide polymorphism, Genetic diversity

## Abstract

**Background:**

*Stenotaphrum secundatum* is an important grass with a rich variety of accessions and great potential for development as an economically valuable crop. However, little is known about the genetic diversity of *S. secundatum*, limiting its application and development as a crop. Here, to provide a theoretical basis for further conservation, utilization, and classification of *S. secundatum* germplasm resources, we used phenotypic and molecular markers (single-nucleotide polymorphisms, SNPs; sequence-related amplified polymorphism, SRAP; inter-simple sequence repeat, ISSR) to analyze the genetic diversity of 49 *S. secundatum* accessions.

**Results:**

Based on seven types of phenotypic data, the 49 *S. secundatum* accessions could be divided into three classes with great variation. We identified 1,280,873 SNPs in the 49 accessions, among which 66.22% were transition SNPs and 33.78% were transversion SNPs. Among these, C/T was the most common (19.12%) and G/C the least common (3.68%). Using 28 SRAP primers, 267 polymorphic bands were detected from the 273 bands amplified. In addition, 27 ISSR markers generated 527 amplification bands, all of which were polymorphic. Both marker types revealed a high level of genetic diversity, with ISSR markers showing a higher percentage of polymorphic loci (100%) than SRAP markers (97.8%). The genetic diversity of the accessions based on SRAP markers (*h* = 0.47, *I* = 0.66) and ISSR markers (*h* = 0.45, *I* = 0.64) supports the notion that the *S. secundatum* accessions are highly diverse. *S. secundatum* could be divided into three classes based on the evaluated molecular markers.

**Conclusions:**

Phenotypic and molecular marker analysis using SNP, SRAP, and ISSR markers revealed great genetic variation among *S. secundatum* accessions, which were consistently divided into three classes. Our findings provide a theoretical basis for the genetic diversity and classification of *S. secundatum*. Our results indicate that SNP, SRAP and ISSR markers are reliable and effective for analyzing genetic diversity in *S. secundatum*. The SNPs identified in this study could be used to distinguish *S. secundatum* accessions.

## Background

*Stenotaphrum* is a perennial Poaceae genus in the Gramineae family. This genus, comprising eight species, is widely distributed in the Pacific Islands, Africa, and the Americas [1]. *Stenotaphrum secundatum* is a warm-season grass species commonly utilized in the turfgrass industry. Compared with other warm-season turfgrasses, *S. secundatum* is known for its tolerance to shade [2], drought [3], and disease [4]. Within *Stenotaphrum*, *S. secundatum* has strong adaptability to various soil conditions and resistance to moisture and flooding, making it particularly suitable for planting in urban regions and in lawns in humid, low-lying areas [5]. In addition, *S. secundatum* exhibits the ability to trail, strong regenerative capacity, and rapid reproduction, suggesting it could be used for soil and water conservation in warm regions and for the greening of barren hills. *S. secundatum* can be easily managed at low cost, supporting its use as a commercial warm-season lawn grass [5]. All these characteristics make *S. secundatum* a valuable turfgrass.

Previous studies of *S. secundatum* have mainly focused on its ecological habits and morphological characteristics. *S. secundatum* has abundant genetic polymorphisms [6, 7], but little is known about the genetic relationships and diversity of *S. secundatum* accessions. A few studies have focused on the genetic diversity of *S. secundatum* germplasm using amplified fragment length polymorphism (AFLP) [8] and simple sequence repeat (SSR) markers [7]. Genovesi et al. [9] used EST-derived microsatellites (EST-SSRs) to identify heterozygosity and evaluate genetic variation in 25 *S. secundatum* lines obtained from ploidy crosses. Yu et al. [10] used 2871 SNPs and 81 SSRs to develop a high-density genetic map of *S. secundatum* and identified putative quantitative trait loci (QTL) related to turf quality.

Molecular markers are excellent tools for exploring the genetic diversity of different plants, especially for classifying species that are difficult to identify using traditional classification methods [11]. Molecular markers are also useful for analyzing the evolutionary relationships between different plant groups [12]. A wide variety of molecular marker types have been employed to evaluate the genetic diversity of grasses, including SNP [13], ISSR [14, 15], and SRAP [16] markers. SNPs refer to single-nucleotide differences in the genomic DNA sequences of different organisms. The advantages of SNPs are that they contain only single-nucleotide variations, are widely distributed, and show high genetic stability [17]. SNPs have been successfully used to analyze the genetic diversity of *Nicotiana tabacum* and *Triticum aestivum* [18, 19]. Li et al. [20] used SNP markers to analyze 59 cabbage varieties and identified 417 SNPs that were used as core markers to construct DNA fingerprints in order to obtain SNP fingerprints. SNPs were also used to identify 105 maize inbred lines by genotyping point mutations [21].

Here, we performed statistical analysis of seven phenotypic traits in a panel of *S. secundatum* accessions and analyzed their genetic diversity using SNP, SRAP, and ISSR markers, elucidating the genetic diversity within *S. secundatum.* We compared genetic differences among accessions from different sources to identify good germplasm resources, providing a theoretical basis for breeding excellent individuals and laying the foundation for the conservation and utilization of *S. secundatum*.

## Results

### *S. secundatum* resources exhibit abundant phenotypic diversity

Analysis of seven phenotypic traits in 49 *S. secundatum* accessions showed that plants from different regions exhibited extensive phenotypic variation. The average coefficient of variation (CV) of the seven phenotypic traits was 15.71%. The CV was highest for height of the erect shoot (25.82%), followed by leaf length of the erect shoot, stolon length, and leaf width of the erect shoot. The variation in leaf color (from yellow-green to dark-green) was small. The CV values for leaf length of the erect shoot and erect shoot height were > 20% (Additional file 1: Table S1).

Principal component analysis (PCA) (Additional file 2: Figure S1) showed that the cumulative contribution of all three principal components was as high as 81.38%, which basically represents the comprehensive variation of all seven traits. The contribution ratio of the first principal component was 56.23%, with the highest input from height of the erect shoot, leaf length of the erect shoot, and length of the stolon, indicating that these three morphological traits are the most important variable factors in the phenotypic diversity of *S. secundatum*. The contribution ratios of the second and third principal components were 15.87 and 9.28%, respectively, with the highest input from leaf width of the erect shoot and turf quality.

We estimated the correlation coefficients between the seven phenotypic traits by correlation analysis (Table 1). There was a positive correlation between leaf length of the erect shoot and stolon length (^*^P < 0.05). Stolon diameter was positively correlated with leaf width of the erect shoot (^**^P < 0.01) and negatively correlated with stolon length (^**^P < 0.01). The height of the erect shoot was positively correlated with leaf length of the erect shoot and stolon length (^**^P < 0.01). There was a highly positive correlation between turf quality and leaf color (^**^P < 0.01). The highest correlation coefficient was 0.696 for height of the erect shoot and leaf length of the erect shoot.

**Table 1:** Analysis of the correlation among phenotypic characteristics of the *S. secundatum* accessions (*n* = 49)

	Leaf width of the erect shoot (cm)	Leaf length of the erect shoot (cm)	Length of the stolon (cm)	Diameter of the stolon (mm)	Height of the erect shoot (cm)	Leaf color	Turf quality
**Leaf width of the erect shoot (cm)**	1						
**Leaf length of the erect shoot (cm)**	0.215	1					
**Length of the stolon (cm)**	−0.186	0.310^a^	1				
**Diameter of the stolon (mm)**	0.567^b^	0.096	−0.426^b^	1			
**Height of the erect shoot (cm)**	0.215	0.696^b^	0.609^b^	−0.084	1		
**Leaf color**	0.137	0.109	−0.113	−0.094	0.066	1	
**Turf quality**	0.161	0.054	−0.037	−0.169	−0.074	0.598^b^	1

Note: ^a^indicates a significant correlation at the 0.05 level (bilateral). ^b^indicates a significant correlation at the 0.01 level (bilateral)

**Fig. 1:**
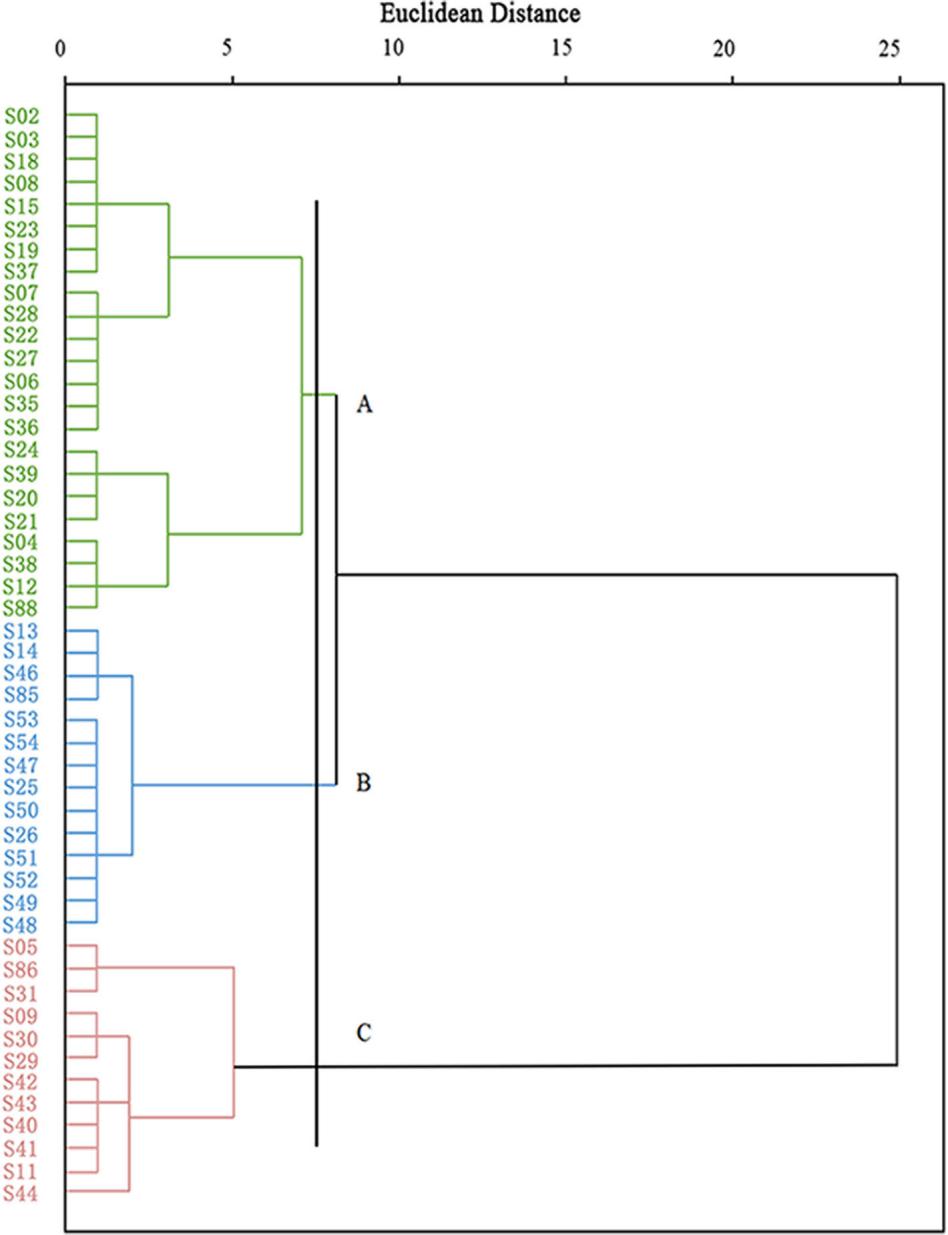
Clustering analysis of *S. secundatum* accessions based on phenotypic characters

Based on these seven phenotypic traits, we performed cluster analysis with SPSS 22.0. Cluster analysis divided the 49 *S. secundatum* accessions into three classes (Classes A, B, and C) at a Euclidean distance of 7.5 (Fig. 1). Class A contained 23 accessions (S02, S03, S18, S08, S15, S23, S19, S37, S07, S28, S22, S27, S06, S35, S36, S24, S39, S20, S21, S04, S38, S12, and S88) with short leaf length of the erect shoot, short height of the erect shoot, and low turf quality. Class B included 14 accessions (S13, S14, S46, S85, S53, S54, S47, S25, S50, S26, S51, S52, S49, and S48) with short leaf length of the erect shoot, short height of the erect shoot, and high turf quality. Class C included 12 accessions with longer leaf length of the erect shoot and higher height of the erect shoot, including three with shorter stolons (S05, S86, and S31) and nine with longer stolons (S09, S29, S30, S42, S43, S40, S41, S11, and S44).

### Genetic diversity and genetic relationships of *S. secundatum* accessions based on SNP analysis

To ensure the quality of the data, we performed quality control on the original data and reduced data noise through data filtering to obtain high-quality clean reads for subsequent analysis (Additional file 1: Table S2). We obtained 396,610,302 reads from the 49 accessions after filtration, with the number of reads per accession ranging from 2,830,814 to 18,264,310 (Additional file 1: Table S3) and averaging 8,094,088. The sequencing quality value (Q30) ranged from 87.45 to 92.24%, with an average value of 89.16%. The GC content obtained by sequencing ranged from 42.07 to 45.35%, with an average value of 43.26%. The Q30 values of all accessions were greater than 80%, indicating that the string error rate was low and the data were of high quality. The GC content was ubiquitous, indicating that the requirements were met. A total of 1,844,338 restriction-site associated DNA tags (RAD-tags) were developed for the 49 *S. secundatum* accessions, and 1,280,873 SNP tags were developed using RAD-tags. These SNPs included 66.22% transition SNPs and 33.78% transversion SNPs (Additional file 2: Figure S2). Among these, C/T was the most common (19.12%) and G/C the least common (3.68%).

**Fig. 2:**
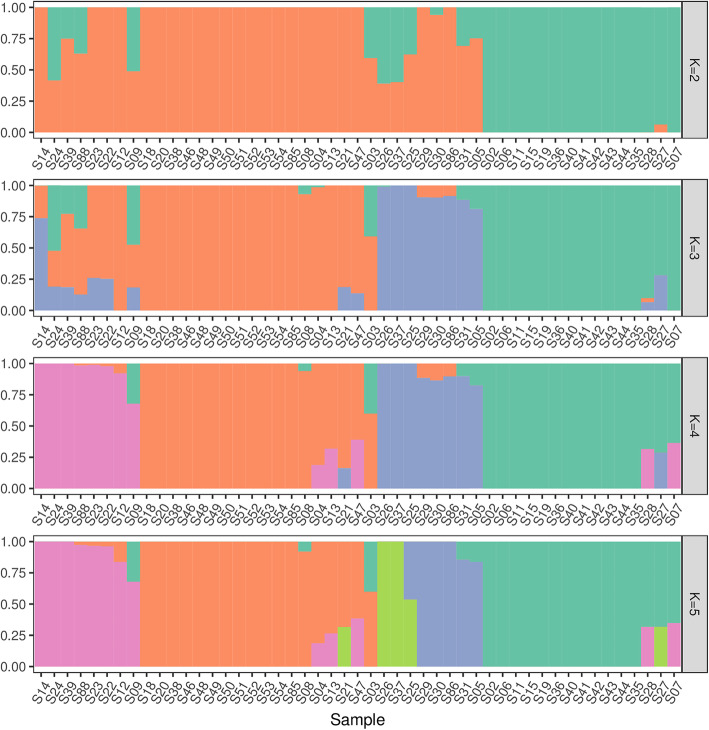
Structural clustering of 49 *S. secundatum* accessions (cultivars) based on SNP data

We used Frappe1.1 to cluster the 49 *S. secundatum* accessions based on the 1,280,873 SNPs in the filtered reads populations (Fig. 2). From K = 2–5, S18, S20, S38, S46, S48, S49, S50, S51, S52, S53, S54, and S85 were always clustered in the same class, indicating that their genetic relationship was relatively close. S02, S06, S11, S15, S19, S36, S40, S41, S42, S43, S44, and S35 were always clustered in the same class, indicating that their genetic relationship was close, whereas S09, S08, S03, S31, S05, and S27 were clearly separated from the other accessions, indicating that their genetic relationship was more distant.

**Fig. 3:**
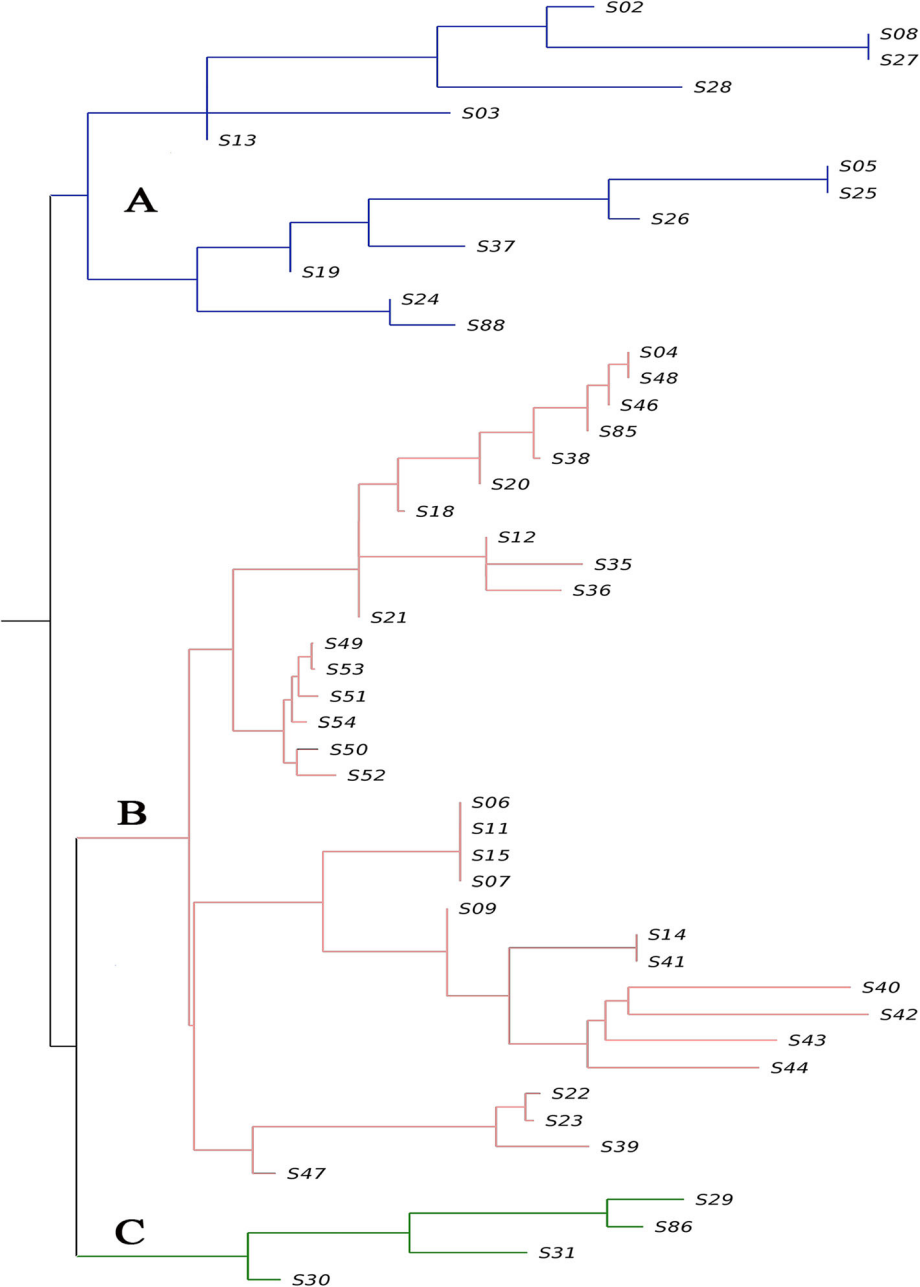
Neighbor-joining clustering of 49 *S. secundatum* accessions (cultivars) based on SNP data

We generated a distance matrix and constructed a clustering diagram of the 49 accessions based on the 1,280,873 SNPs in the filtered population (Fig. 3). The 49 *S. secundatum* accessions were divided into three classes in the clustering diagram. Class A contained 13 accessions, including four accessions from Hainan (S02, S28, S03, and S24), four from Guangxi (S08, S05, S37, and S19), two from Yunnan (S27 and S13), one from Fujian (S25), one from Guangdong (S26), and one from the Vanuatu (S88). Class B contained 32 accessions, including nine accessions from Hainan (S04, S48, S85, S20, S12, S36, S21, S11, and S39), eight from Guangxi (S38, S18, S09, S41, S40, S42, S43, and S44), eight from Fujian (S49, S53, S51, S54, S50, S52, S22, and S23), four from Guangdong (S46, S35, S06, and S07), two from Yunnan (S15 and S14), and one from Jiangsu (S47). Class C contained four accessions, including two accessions from Hainan (S29 and S30), one from Guangdong (S86), and one from Yunnan (S31).

PCA (Additional file 2: Figure S3) of the 49 accessions revealed that S40, S41, S42, S43, and S44 were clustered together and were significantly separated from the other accessions. S02, S27, and S28 were clustered together and separated from the other accessions. S03 was grouped in a single class, whereas most accessions were grouped together. The results of PCA were congruent with the results of cluster analysis and structural clustering in that they clearly showed the relationships among accession pedigrees.

### Genetic diversity and genetic relationships of *S. secundatum* accessions based on SRAP analysis

We amplified *S. secundatum* DNA using 28 pairs of SRAP polymorphic primers and consistently obtained 273 clear, bright bands, including 267 polymorphic bands, with a polymorphism rate of 97.8%. Each pair of primers amplified six to 13 bands (Additional file 1: Table S4). Additional file 2: Figure S4 shows the spectra of amplified bands from 46 *S. secundatum* accessions obtained using Primer EM14-ME4. The amplified DNA bands ranged from 200 to 400 bp, and the ratio of polymorphic bands amplified by each pair of primers was 87.5–100%. Thus, the SRAP markers in *S. secundatum* showed abundant polymorphism. We used PopGen32 to analyze the genetic diversity of the 46 *S. secundatum* accessions. The average Nei’s genetic diversity (*h*) value of the accessions was 0.47, and the average Shannon’s information index (*I*) was 0.66 (Additional file 1: Table S5). Both values were relatively high, indicating that there was great variation and rich genetic diversity among different *S. secundatum* germplasms. The genetic similarity coefficients of the materials ranged from 0.36 to 0.84 (Additional file 1: Table S6). The genetic similarity coefficient between S40 and S43, which were both from Guangxi, was 0.84, indicating that their relationship was the closest. The genetic similarity coefficient between S09 from Hainan and S26 from Guangdong was the smallest, indicating that they had a distant genetic relationship.

**Fig. 4:**
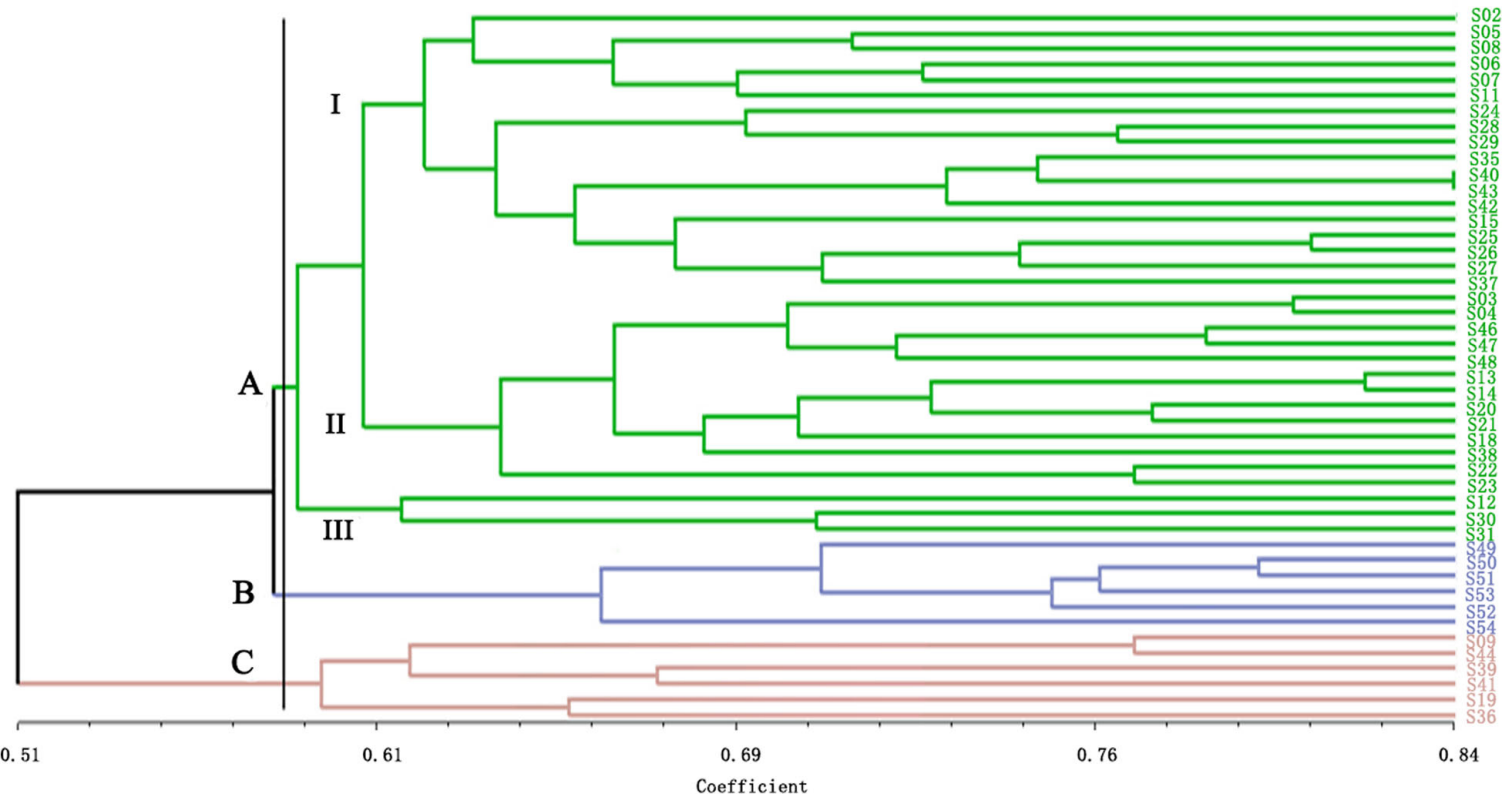
UPGMA clustering of 46 *S. secundatum* accessions based on SRAP data

UPGMA (Unweighted Pair Group Method with Arithmetic means) clustering (Fig. 4) of the 46 *S. secundatum* accessions showed that S02 from Hainan and S36 from Fujian were the furthest apart in the clustering diagram, with the 44 remaining accessions distributed between them. UPGMA clustering also divided the 46 accessions into three classes (Class A, B, and C), with a similarity coefficient of 0.59. Class A contained 34 *S. secundatum* accessions, which were all *S. secundatum* var. ‘Variegatum’ (S04, S48, S20, S21, S38, and S12). Class A was divided into subclasses I, II, and III. Subclass I contained 18 accessions, including six accessions from Guangxi (S05, S08, S40, S42, S43, and S37), five from Hainan (S02, S11, S24, S28, and S29), four from Guangdong (S06, S07, S35, and S26), two from Yunnan (S15 and S27), and one from Fujian (S25). Subclass II contained 13 accessions, including five accessions from Hainan (S03, S04, S48, S20, and S21), two from Yunnan (S13 and S14), two from Guangxi (S18 and S38), two from Fujian (S22 and S23), one from Guangdong (S46), and one from Jiangsu (S47). Subclass III contained two accessions from Hainan (S12 and S30) and S31 from Yunnan. Class B contained six accessions, which were all from Fujian (S49, S50, S51, S53, S52 and S54). Class C contained six accessions (S09, S44, S39, S41, S19 and S36), including four accessions from Hainan and two from Guangxi.

### Genetic diversity and genetic relationships of *S. secundatum* accessions based on ISSR analysis

We also analyzed the genetic diversity of the 46 *S. secundatum* accessions using 27 ISSR primers. In total, 527 bands were amplified, which all were polymorphic. The average number of bands amplified by each primer was 19.52, with 14 to 25 bands amplified per primer (Additional file 1: Table S7), indicating that *S. secundatum* shows abundant ISSR polymorphism. Additional file 2: Figure S5 shows the spectra of amplified bands from the 46 *S. secundatum* accessions obtained using Primer ISSR889. PopGen32 was used to analyze their genetic diversity. The *h* value of the 46 accessions was 0.45, and the *I* value was 0.64 (Additional file 1: Table S8). Both values are relatively high, indicating that there was great variation and rich genetic diversity in the *S. secundatum* germplasm. These results indicate that ISSR markers could be successfully used to reveal the genetic differences among germplasm resources.

**Fig. 5:**
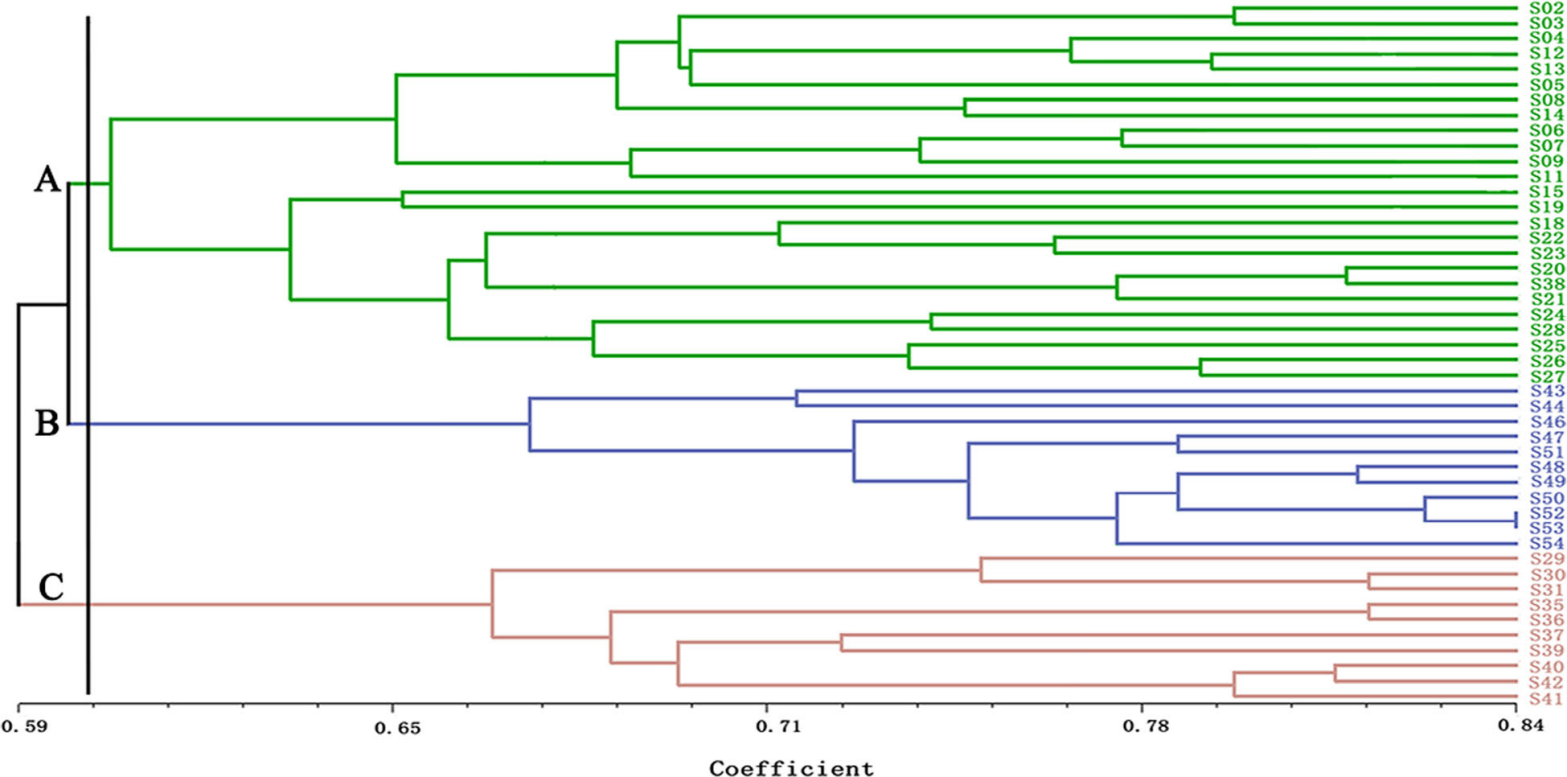
UPGMA clustering of 46 *S. secundatum* accessions based on ISSR data

The genetic similarity coefficient of *S. secundatum* was 0.50–0.84 (Additional file 1: Table S9), indicating that the materials had rich genetic diversity. The genetic similarity coefficient between S52 and S53 was 0.84, indicating that their relationship was the closest. The genetic similarity coefficient between S04 from Hainan and S35 from Guangdong was 0.50, indicating that they had a distant genetic relationship. UPGMA clustering of the 46 *S. secundatum* accessions showed that S02 and S41 were the furthest apart in the clustering diagram, with the 44 remaining germplasm resources distributed between them (Fig. 5). UPGMA clustering also divided the 46 accessions into three classes (Class A, B, and C), with a similarity coefficient of 0.60. Class A contained 25 accessions, including nine accessions from Hainan (S02, S03, S04, S11, S12, S24, S28, S20, and S21), six from Guangxi (S05, S08, S09, S18, S19, and S38), four from Yunnan (S13, S14, S15, and S27), three from Guangdong (S06, S07, and S26), and three from Fujian (S22, S23, and S25). Class B contained six accessions (S49, S50, S51, S53, S52, and S54), and Class C contained 10 accessions, including four accessions from Guangxi (S37, S40, S41, and S42), four from Hainan (S29, S30, S36, and S39), one from Yunnan (S31), and one from Guangdong (S35).

## Discussion

### *S. secundatum* accessions show high levels of variation

The phenotypes of the *S. secundatum* accessions examined in the current study covered a larger range of variation than those examined previously [22]. Indeed, the phenotypes of the *S. secundatum* accessions showed great variability, providing a basis for the multiple uses of *S. secundatum*. Among the 49 *S. secundatum* germplasm resources examined here, the grass turf quality was high for many accessions (such as S47, S48, S49, S50, S51, S52, S53, and S54), indicating that they could be used as turfgrass breeding materials. Various *S. secundatum* accessions (such as S09, S11, S40, S41, and S44) had long and wide leaves and long stolon internodes, indicating that they could be used as breeding materials for solid soil slope protection in lawns or pastures. In summary, the availability of abundant *S. secundatum* resources could facilitate the selection and breeding of fine turfgrasses with broad applications and high economic potential.

From the perspective of modern genetics, species with higher genetic diversity will have a wider natural distribution and stronger environmental adaptability, survivability, and evolutionary potential than other species. Genetic variation is required for a species to adapt to environmental changes [23]. Therefore, evaluating genetic diversity is an important part of germplasm identification and collection. In the current study, the ISSR markers provided more informative data than SRAP markers. Similar results have been obtained for *Elymus pastures* [24], tobacco [25], and *Grifola frondosa* [26] using ISSR and SRAP molecular markers. Overall, though our SRAP and ISSR markers returned higher *S. secundatum* species diversity numbers than those previously reported values for other species, such as *Pennisetum* (*h* = 0.3306, *I* = 0.5017) obtained using SRAP markers [27] and *Stylosanthes* (*h =* 0.2279, *I* = 0.3553) obtained using ISSR markers [28]. We conclude that SRAP and ISSR markers can effectively used to reveal the polymorphism between *S. secundatum* accessions, with high labeling efficiency.

The SNPs uncovered in this study represent the largest number of SNPs for *S. secundatum* identified to date, revealing large intraspecific differences. These types of SNPs could be used for population-level evolutionary analysis. However, the 1,280,873 SNPs obtained in the current study are not sufficient for this purpose. We will continue to collect more germplasm and analyze the association between phenotypes and SNPs to identify variations associated with important traits. These SNP markers could also provide a basis for the development of SNP markers in other species of *Stenotaphrum.*

### Analyses based on phenotypic traits and SNP, SRAP, and ISSR markers divide *S. secundatum* from China into three classes

In the present study, the *S. secundatum* accessions clustered into three classes based on seven phenotypic traits. In addition to using phenotypic markers, we used three types of molecular markers (SNP, SRAP, and ISSR markers) to comprehensively analyze the genetic diversity of *S. secundatum.* Cluster analysis based on phenotypes and on three types of molecular markers produced consistent results, with some minor differences: in all cases, the accessions were clustered into three classes, and seven accessions of *S. secundatum* (S02, S03, S04, S08, S24, S27, and S28) were clustered into a single group. In our phenotypic and molecular marker clustering of the 49 *S. secundatum* accessions, most accessions from the same region were clustered into one group, and only a few were placed into different groups, perhaps reflecting the spread of introduced genes or the infiltration of certain genes [29]. Clustering using all three markers classified nine *S. secundatum* accessions from Fujian into two groups. Four accessions from Guangdong (S06 and S07) and Fujian (S23 and S22) were clustered together and showed similar phenotypes, including low values for height of the erect shoot and leaf width of the erect shoot. Perhaps because these two provinces are adjacent, human activities and natural factors caused these accessions to spread and reproduce in both provinces. Similar observations have been made for other germplasms [30, 31].

Our clustering based on phenotypic and molecular markers produced similar results, indicating that the phenotypic markers and different molecular markers performed convergently. This consistency points to the reliability of our current classification of the genetic diversity of *S. secundatum* based on phenotypic traits. Similarly, 300 *Gossypium hirsutum* accessions were divided into three classes based on phenotypic traits and SSR markers [32], and 16 *Tagetes patula* accessions were placed into three classes based phenotypic traits and ISSR markers [33]. These results from a range of species indicate that classification based on phenotypic traits is suitable for genetic diversity analysis.

Clustering can also be used to determine the genetic diversity and genetic relationships between germplasm from different regions. Due to difficulties in obtaining samples, most of our experimental germplasm resources were from China. In another study, AFLP markers were used to detect genetic variants of the *S. secundatum* cultivar ‘Raleigh’ collected from sod farms across the southern United States [8]. This analysis revealed separation between original stocks of ‘Raleigh’ and some commercial accessions. Little is known about the genetic relationships and diversity of *S. secundatum* accessions. The genetic information obtained in the current study could be a very useful for evaluating the genetic diversity in *S. secundatum* and for future molecular marker-assisted breeding programs.

## Conclusions

Seven phenotypes of 49 *S. secundatum* were comprehensively analyzed and clustered. The phenotypes of *S. secundatum* showed large variation. We used RAD-seq technology to evaluate the *S. secundatum* population and performed simplified genome sequencing, SNP detection, and population-level genetic evolutionary analysis, which indicated that RAD-seq technology could successfully be utilized for *S. secundatum*. We obtained 396,610,302 reads from the 49 *S. secundatum* accessions by RAD-seq and developed 1,844,338 RAD-tags based on the read information and 1,280,873 SNPs based on the RAD-tags. Overall, our analyses of 49 *S. secundatum* accessions from China, including phenotypic analysis and DNA molecular marker analysis (SNP, SRAP, and ISSR markers), showed that *S. secundatum* could be divided into three classes. We systematically analyzed the genetic diversity of *S. secundatum* germplasm resources to investigate their genetic relationships, providing a theoretical basis for the classification of *S. secundatum* and laying a scientific foundation for the conservation and utilization of *S. secundatum* germplasm resources.

## Methods

### Plant materials and phenotypic analysis

Forty-nine *S. secundatum* accessions, which were obtained from China and the South Pacific, were analyzed in this study (Additional file 1: Table S10). Of these, 48 *S. secundatum* accessions were collected from different regions of China and one was collected from the Vanuatu, South Pacific. The accessions were grown in Haidian Agricultural Base in Hainan University [19° 31′ N, 109° 34′ E] with unified management. The base is located in the northern edge of the tropics, with a tropical monsoon climate. The annual average temperature is approximately 22–26 °C and the annual illumination is approximately 1750–2650 h. The light rate was 50–60%, and the solar radiation intensity was 14,012.2–53,587.4 Lux. There was abundant rainfall, with average annual precipitation of 1815 mm.

The *S. secundatum* accessions were cultured in a plot with an area of 1 m^2^. Routine management was performed, including watering, fertilization, regular pruning, and cleaning of creeping branches. Young leaves were collected from the 49 accessions, stored in an icebox, and subjected to laboratory analysis in a timely manner.

The leaf width of the erect shoot, leaf length of the erect shoot, stolon length, stolon diameter, height of the erect shoot, leaf color, and turf quality of the 49 *S. secundatum* accessions were measured as described by Liao et al. and Liu et al. [34, 35]. A vernier caliper was used to measure the width and length of the fourth mature leaf from the top to the base of the erect shoot (Leaf width and length of the erect shoot) and the internode length and diameter of the fourth stem segment of stolon from top to base (Length and diameter of the stolon). A ruler was used to measure the height of the erect shoot. The leaf color was scored (and repeated three times) based on the following criteria: one point for withered yellow lawn or bare land; one to three points for more dead leaves and less green tissue; five points for more green plants and a small amount of dead leaves; five to nine points for lawns from yellow-green to dark green. Based on the indicators of leaf color, texture, density, and uniformity, turf quality was evaluated according to the 5-level standard of external character observations.

SPSS 22.0 was used to calculate the correlation between the seven phenotypic traits and to cluster the *S. secundatum* accessions based on the phenotypic data. The differences between the phenotypes of different materials were expressed as coefficient of variation, $CV\left(\%\right)=\left(S/\overline{X}\right)\times 100$, where S is the standard deviation and $\overline{X}$ is the average value of the individual trait. The F-values were calculated to examine the significance of the observed variation.

### DNA extraction and SNP analysis

DNA was extracted from the 49 leaf samples using a Plant Genomic DNA Kit (DP305–03; Tiangen, Beijing, China). The following steps were used to create a library by enzyme digestion: an equal amount of genomic DNA (100 ng) was incubated with restriction enzymes in a 50 μl reaction system at 37 °C for 1 h, followed by incubation at 65 °C for 20 min to inactivate the endonuclease. Each enzyme digestion product was ligated to an adapter with a restriction enzyme sticky end and a barcode label, incubated at 20 °C for 30 min, and inactivated at 65 °C for 20 min. The accession containing the adapter was mixed, and the DNA mixture was cut into small bands of ~ 380 bp using a Covaris DNA ultrasonic interrupter. The fragmented DNA was purified using a Qiagen PCR purification kit (Qiagen, Germany), dissolved in 50 μL EB, and stored at 4 °C. The purified DNA was used to construct an Illumina sequencing library with a TruSeq Nano DNA Library Preparation Kit (Illumina, USA), which was sequenced on the Illumina HiSeq 4000 platform (Illumina, USA).

To ensure the quality of the sequences, we performed quality control on the original data prior to analysis and reduced data noise through data filtering. The filtering steps included the following: remove reads < 50 nt long after trimming 3′ sequencing adapters; remove reads with an unknown base (N) ratio > 10%, and remove low-quality reads (number of bases with quality value SQ ≤ 20 accounting for more than 50% of the entire read).

Q30 and GC content were analyzed using BWA (v0.7.15-r1140) software [36] to ensure the quality of the sequencing data. The reads data from the 49 different samples were clustered, and SAMtools (v1.3.1) software [37] was used to classify and splice reads, connect the reads, and build RAD-tags. GATK (v3.5) software [38] was used for mutation detection: GATK HaplotypeCaller was used to perform SNP calling on each accession, and GATK GenotypeGVCFs was used to merge the SNP results (VCF) from the 49 samples and convert the SNP from the pseudogenomic position back to the original rad sequence position to obtain SNPs. Frappe1.1 software [39] was used to identify the genetic structure of the 49 samples and group lineage information. MEGA 6.0 [40] software was used to calculate the genetic distance with the Kimura distance model and to construct dendrograms with the adjacency method (NJ, Neighbor-Joining). EIGENSOFT 7.2.0 software [41] was used for PCA.

### SRAP-PCR amplification

The primers were screened based on the principle proposed by Li and Quiro [42] and synthesized by Yingwei Jieji Trading Company (Shanghai). Of the 400 primer pairs examined, 28 pairs generating clear, bright, polymorphic bands were selected and successfully amplified in 46 *S. secundatum* accessions (Additional file 1: Table S11). The reaction system used for SRAP-PCR amplification included 2.0 μl 5× buffer, 2.0 mM Mg^2+^, 200 μM dNTP, 0.3 μM primers, 1.0 U Taq polymerase, 100 ng DNA, and ddH_2_O to a final volume of 10 μL. The amplification program was as follows: step 1, pre-denaturation at 94 °C for 5 min, denaturation at 94 °C for 1 min, annealing at 33 °C for 1 min, extension at 72 °C for 60 s; step 1 was repeated for 5 cycles; step 2, denaturation at 94 °C for 1 min, annealing at 55 °C for 1 min, extension at 72 °C for 60 s; step 2 was repeated for 35 cycles; step 3, extension at 72 °C for 7 min. The final PCR product was stored at 4 °C. After the amplification reaction, the 10 μl amplified product was separated on a 10% non-denaturing polyacrylamide gel using the electrode buffer 1× TBE for rapid silver staining detection. The DNA bands were fixed in the gel in 180 ml ddH_2_O + 20 ml ethanol + 1 ml acetic acid glacial for 12 min with shaking. The gel was stained in 300 ml ddH_2_O + 0.6 g AgNO_3_ for 12 min with shaking. After staining, the gel was rinsed for three times with ddH_2_O and incubated in 300 mL ddH_2_O + 60 μL 10% sodium thiosulfate for 30 s, followed by developing solution (300 ml ddH_2_O + 4.5 g NaOH + 3 ml formaldehyde) until clear bands were obtained. The gel was observed on a film observation lamp and photographed.

### ISSR amplification

ISSR molecular marker [43] analysis was performed using 100 ISSR primers described by the University of British Columbia (S801–S900) and synthesized by Yingwei Jieji Trading Company (Shanghai). The 100 primers were screened by amplifying DNA from two random *S. secundatum* accessions, and 27 primers producing clear amplification bands with good polymorphism were selected (Additional file 1: Table S12). A previously described reaction system for ISSR PCR amplification (10 μL) [44] was used with some modifications: 1.5 μL 10× buffer (100 mM Tris–HCl pH 8.3, 500 mM KCl, 15 mM MgCl_2_), 200 μM dNTPs, 0.3 μM primers, 1.0 U Taq polymerase, 100 ng DNA, and 5.1 μL ddH_2_O was used for amplification. The amplification program was as follows: step 1, pre-denaturation at 94 °C for 5 min; step 2, denaturation at 94 °C for 1 min, annealing at 55–59 °C for 1 min, extension at 72 °C for 90 s; step 2 was repeated for 45 cycles; step 3, extension at 72 °C for 7 min. The PCR product was stored at 4 °C. The 10% gel was prepared as follows: 40 ml ddH_2_O, 20 ml 5× TBE, 20 ml 40% acrylamide, 90 μL TEMED, 900 μL 10% AP. A 3.1–3.3 μL aliquot of each accession was subjected to electrophoresis for 3.5 h at 120–140 V and 100 mA.

### SRAP and ISSR data analysis

The presence of a band was assigned a value of “1” and its absence was assigned a value of “0”; unclear bands were not included in the analysis. The statistical results were expressed as a 0–1 matrix, and the data were analyzed with EXCEL, PopGen32 [45], and NTSYS [46]. EXCEL was used to integrate the raw data matrices of SRAP and ISSR markers. PopGen32 was used to calculate the number of polymorphic bands, the percentage of polymorphic loci, the Nei’s Gene Diversity, and the Shannon Information Index. NTSYS was used to calculate genetic similarities and to build dendrograms using both the UPGMA [47] and neighbor-joining clustering procedures. Nei’s genetic similarity coefficients [48] and cluster analysis were used to determine the genetic distance and genetic diversity among the various *S. secundatum* accessions*.*

## Supplementary information

**Additional file 1: Table S1.** Phenotypic characteristics of *S. secundatum* (*n* = 49). **Table S2.** Summary of RAD-seq. **Table S3.** Statistics for raw data (RAD-seq) from the 49 *S. secundatum* accessions (cultivar). **Table S4.** Detailed information on polymorphism revealed by 28 SRAP primer pairs. **Table S5.** Genetic diversity parameters (h and I) of 46 *S. secundatum* accessions based on SRAP markers. **Table S6.** Genetic similarity coefficients between 46 *S. secundatum* accessions based on SRAP data. **Table S7.** Primer sequences and amplification results of ISSR analysis. **Table S8.** Genetic diversity parameters (h and I) of 46 *S. secundatum* accessions based on ISSR markers. **Table S9.** Genetic similarity coefficients between 46 *S. secundatum* accessions based on ISSR data. **Table S10.** Origins of the 49 *S. secundatum* accessions examined in this study. **Table S11.** Primer sequences used for SRAP analysis of *S. secundatum.* **Table S12.** Primer sequences used for ISSR analysis of *S. secundatum.*

**Additional file 2: Figure S1.** Principal component analysis of 49 *S. secundatum* accessions*.* The contribution ratios of the first principal component and the second principal component were 56.23 and 15.87%, respectively. Note: The codes correspond to those listed in Additional file 1: Table S10. **Figure S2.** Statistics of SNP types in 49 *S. secundatum* accessions. **Figure S3.** PCA of 49 *S. secundatum* accessions (cultivars) based on SNP data. **Figure S4.** Sample amplification profiles of 46 *S. secundatum* germplasms based on SRAP primers EM14-ME4. M: 100 bp marker. Note: The codes correspond to those listed in Additional file 1: Table S10. **Figure S5.** Sample amplification profiles of 46 *S. secundatum* germplasms based on ISSR primer ISSR889. M: 100 bp marker. Note: The codes correspond to those listed in Additional file 1: Table S10.

## Data Availability

All relevant datasets supporting the conclusions of this article are available within the article and in Additional files. The raw sequence data reported in this paper have been deposited in the Genome Sequence Archive (Genomics, Proteomics and Bioinformatics, 2017) in BIG Data Center under accession number CRA002158 and are publicly accessible at http://bigd.big.ac.cn/gsa/s/8uHDmiy8. The SNP data are available in VCF under accession number GVM000057 and are publicly accessible at http://bigd.big.ac.cn/gvm/getProjectDetail?project=GVM000057.

## Abbreviations

AFLP: Amplified fragment length polymorphism
CV: Coefficient of variation
EST-SSR: EST-derived microsatellites
*h*: Nei’s gene diversity
*I*: Shannon information index
ISSR: Inter-simple sequence repeat
NJ: Neighbor-Joining
PCA: Principal component analysis
QTL: Quantitative trait loci
RAD-Seq: Restriction-site associated DNA sequencing
RAD-tags: Restriction-site associated DNA tags
SNPs: Single-nucleotide polymorphisms
SRAP: Sequence-related amplified polymorphism
SSR: Simple sequence repeat
UPGMA: Unweighted pair group method with arithmetic means
