# Supplementary material for: Phenotypic and molecular marker analysis uncovers the genetic diversity of the grass Stenotaphrum secundatum
Source: BMC Genet. 2020 Aug 12;21:86. doi: 10.1186/s12863-020-00892-w (PMC7425169; doi:10.1186/s12863-020-00892-w)
Supplement: Supplementary file 1 — Additional file 1: Table S1. Phenotypic characteristics of S. secundatum (n = 49). Table S2. Summary of RAD-seq. Table S3. Statistics for raw data (RAD-seq) from the 49 S. secundatum accessions (cultivar). Table S4. Detailed information on polymorphism revealed by 28 SRAP primer pairs. Table S5. Genetic diversity parameters (h and I) of 46 S. secundatum accessions based on SRAP markers. Table S6. Genetic similarity coefficients between 46 S. secundatum accessions based on SRAP data. Table S7. Primer sequences and amplification results of ISSR analysis. Table S8. Genetic diversity parameters (h and I) of 46 S. secundatum accessions based on ISSR markers. Table S9. Genetic similarity coefficients between 46 S. secundatum accessions based on ISSR data. Table S10. Origins of the 49 S. secundatum accessions examined in this study. Table S11. Primer sequences used for SRAP analysis of S. secundatum. Table S12. Primer sequences used for ISSR analysis of S. secundatum. [file 12863_2020_892_MOESM1_ESM.zip › Table S1-5-Table S7-8Table S1-5-Table S10-12-20200718.pdf]

Table S1 Phenotypic characteristics of *S. secundatum* (n = 49)

Accession NO.	Leaf width of the erect shoot ((cm) ±SD)	Leaf length of the erect shoot ((cm) ±SD)	Length of the stolon ((cm) ±SD)	Diameter of the stolon ((mm) ±SD)	Height of the erect shoot ((cm) ±SD)	Leaf color	Turf quality
S2	0.77±0.11	8.66±2.11	7.07±1.09	2.47±0.34	9.41±2.80	5.67±0.47	6.00±0.82
S3	0.89±0.22	7.91±1.58	7.56±1.11	2.64±0.29	9.25±1.49	5.67±0.47	6.33±0.47
S4	0.85±0.07	7.48±2.23	7.13±1.20	2.87±0.34	7.54±2.32	1.00±0.00	1.00±0.00
S5	0.89±0.11	12.37±2.11	6.87±1.81	2.55±0.49	12.68±2.37	3.67±0.47	6.67±0.47
S6	0.57±0.07	5.29±0.81	11.17±1.35	2.28±0.23	8.06±1.79	3.00±0.00	6.00±0.00
S7	0.66±0.04	6.53±0.75	9.68±1.68	2.04±0.42	10.35±2.15	3.00±0.00	5.67±0.47
S8	0.82±0.09	8.28±1.03	7.08±1.59	3.14±0.29	10.5±1.13	7.33±0.47	5.33±0.47
S9	0.91±0.03	10.4±2.35	11.00±2.06	2.56±0.25	10.83±2.10	5.33±0.47	5.33±0.47
S11	0.83±0.10	11.23±3.51	14.31±1.23	2.53±0.17	13.22±2.76	5.67±0.47	5.00±0.00
S12	0.92±0.19	7.07±1.60	2.91±0.31	2.68±0.19	7.55±2.44	1.00±0.00	1.67±0.47
S13	1.03±0.11	7.81±1.87	4.71±1.34	3.07±0.17	9.48±1.32	6.67±0.47	5.00±0.00
S14	1.04±0.17	7.17±1.69	3.52±1.32	2.63±0.29	9.71±1.14	6.67±0.47	4.67±0.47
S15	0.80±0.03	7.01±1.05	7.18±1.67	2.58±0.21	8.97±2.06	4.67±0.47	5.67±0.47
S18	0.90±0.30	8.12±1.50	8.91±1.55	2.60±0.31	8.67±1.66	6.67±0.47	6.00±0.00
S19	0.80±0.10	8.01±1.56	6.12±1.09	2.66±0.46	9.06±2.17	3.00±0.00	5.67±0.47
S20	0.80±0.11	9.35±2.63	6.06±2.34	3.45±0.31	10.29±1.75	1.00±0.00	1.00±0.00
S21	0.84±0.16	8.73±1.94	6.25±1.79	3.26±0.47	13.14±2.67	1.33±0.47	2.67±0.47
S22	0.76±0.15	5.11±0.92	7.77±2.46	2.37±0.46	7.61±1.96	4.00±0.00	6.00±0.00
S23	0.82±0.12	7.92±1.80	7.09±0.98	2.72±0.27	8.19±1.75	4.00±0.00	4.67±0.47

S24	0.67±0.16	8.71±2.13	7.04±0.89	2.84±0.23	10.83±2.18	5.00±0.00	3.67±0.47
S25	1.04±0.11	7.73±2.40	3.65±1.89	3.10±0.28	7.90±1.77	6.00±0.00	6.67±0.47
S26	0.95±0.11	6.51±2.12	5.28±2.20	3.11±0.29	8.13±2.78	6.67±0.47	7.00±0.00
S27	0.54±0.03	5.25±0.60	8.70±1.78	2.22±0.25	8.08±1.58	4.33±0.47	6.00±0.00
S28	0.95±0.10	7.46±2.20	9.54±1.23	2.38±0.43	9.81±2.73	3.67±0.47	6.00±0.00
S29	0.43±0.04	10.06±2.54	10.59±1.27	1.53±0.08	11.23±2.17	5.67±0.47	6.00±0.00
S30	0.39±0.05	9.17±1.78	12.05±1.04	1.70±0.15	12.45±4.35	6.67±0.47	5.67±0.47
S31	0.79±0.10	13.97±3.48	7.50±1.18	2.63±0.25	14.65±3.52	5.67±0.47	6.33±0.47
S35	0.56±0.06	5.12±1.10	10.13±1.89	2.35±0.29	7.64±2.14	3.33±0.47	5.67±0.47
S36	0.53±0.03	6.49±2.11	10.28±0.94	2.24±0.10	7.59±1.29	3.33±0.47	4.67±0.47
S37	0.81±0.06	8.56±2.17	5.91±1.80	3.46±0.18	9.74±2.16	4.67±0.47	6.33±0.47
S38	0.85±0.27	6.66±1.98	5.33±2.29	3.10±0.28	8.29±2.53	1.00±0.00	1.33±0.47
S39	0.71±0.08	8.88±3.39	8.50±1.42	2.99±0.38	9.86±4.59	4.00±0.00	2.00±0.00
S40	0.85±0.11	10.41±3.18	14.89±1.04	2.42±0.25	12.56±3.60	2.67±0.47	4.67±0.47
S41	0.86±0.15	9.42±3.58	13.79±1.40	2.59±0.13	16.02±5.02	3.00±0.00	5.00±0.00
S42	0.77±0.14	8.14±2.19	13.7±1.77	2.68±0.22	11.24±3.70	3.00±0.00	5.33±0.47
S43	0.75±0.18	10.8±3.60	13.33±2.62	2.71±0.16	9.75±4.00	3.33±0.47	5.33±0.47
S44	1.40±1.58	8.15±2.43	15.1±1.64	2.68±0.19	19.07±3.96	5.67±0.47	5.67±0.47
S46	1.06±0.08	9.39±2.65	4.24±1.36	3.00±0.17	10.91±2.47	6.00±0.82	6.00±0.82
S47	0.74±0.12	7.39±1.71	4.52±1.44	2.46±0.27	6.66±2.14	5.00±0.00	7.33±0.47
S48	1.05±0.22	7.47±2.28	3.83±1.51	3.07±0.33	6.65±1.52	1.00±0.00	7.33±0.47
S49	0.83±0.07	8.18±1.90	6.23±1.34	2.92±0.18	5.86±2.41	6.00±0.00	7.33±0.47
S50	0.89±0.06	7.88±2.42	4.85±1.25	2.95±0.26	8.49±2.90	6.00±0.00	7.00±0.00
S51	0.79±0.18	5.62±1.60	4.99±1.19	2.84±0.50	5.86±1.52	6.00±0.00	7.00±0.82
S52	0.83±0.08	6.18±1.50	4.14±1.44	2.77±0.40	6.29±2.35	5.67±0.47	7.00±0.00

S53	0.81±0.14	6.85±1.89	4.40±1.22	2.63±0.34	7.58±2.02	5.67±0.47	8.33±0.47
S54	0.87±0.07	6.35±1.09	4.30±2.11	2.76±0.41	7.92±2.24	6.00±0.00	7.33±0.47
S85	1.03±0.11	9.24±2.62	1.70±0.42	2.64±0.31	8.68±4.35	6.33±0.47	7.00±0.82
S86	0.86±0.07	11.01±2.83	7.39±0.74	2.96±0.38	15.49±5.99	5.00±0.00	5.67±0.47
S88	0.43±0.12	3.14±0.43	2.90±0.11	2.29±0.02	6.65±1.03	3.67±0.47	1.33±0.47
$\overline{X}$	0.81	8.05	7.85	2.68	9.72	4.48	5.35
S	0.14	2.02	1.44	0.28	2.51	0.28	0.36
CV%	17.28	25.09	18.34	10.45	25.82	6.25	6.73
F	3.88^{**}	6.53^{**}	39.32^{**}	13.97^{**}	7.93^{**}	4.64^{**}	4.68^{**}

Note: $\overline{X}$: Mean; S: Standard deviation; F: F value; CV%: Coefficient of variation, CV%=(S/ $\overline{X}$)∗100; **: $p<0.01$.

Table S2 Summary of RAD-seq

Sample	Clean Reads Num	HQ Clean Reads Num(%)	Read Length	Adapter(%)	Low Quality(%)
S02	7745092	7461350 (96.34%)	150/144	608544 (10.48%)	56056 (0.72%)
S03	14578880	14191686 (97.34%)	150/144	1069533 (9.78%)	159372 (1.09%)
S04	7914674	7875834 (99.51%)	150/144	83559 (1.4%)	3094 (0.04%)
S05	5890076	5871930 (99.69%)	150/144	46347 (1.05%)	2318 (0.04%)
S06	5412370	5391786 (99.62%)	150/146	48999 (1.2%)	1916 (0.04%)
S07	6740126	6710640 (99.56%)	150/146	68676 (1.36%)	2414 (0.04%)
S08	8864524	8816270 (99.46%)	150/146	103416 (1.56%)	2824 (0.03%)
S09	6797578	6772506 (99.63%)	150/146	64353 (1.26%)	2362 (0.03%)
S11	5729758	5705944 (99.58%)	150/145	55851 (1.3%)	2068 (0.04%)
S12	7668930	7625422 (99.43%)	150/145	92808 (1.61%)	2686 (0.04%)
S13	7462090	7422216 (99.47%)	150/145	85911 (1.53%)	3008 (0.04%)
S14	7017510	6994242 (99.67%)	150/143	58302 (1.1%)	2558 (0.04%)
S15	13592258	13574522 (99.87%)	150/143	72990 (0.71%)	4994 (0.04%)
S18	6610880	6579418 (99.52%)	150/142	70533 (1.42%)	2438 (0.04%)
S19	6925098	6902662 (99.68%)	150/142	58137 (1.12%)	2806 (0.04%)
S20	4662800	4470052 (95.87%)	150/142	347823 (9.94%)	15444 (0.33%)
S21	8706594	8661892 (99.49%)	150/142	96432 (1.48%)	2848 (0.03%)
S22	8975918	8897560 (99.13%)	150/144	34071 (0.5%)	75520 (0.84%)
S23	8067888	7785116 (96.50%)	150/144	427020 (7.06%)	92604 (1.15%)
S24	11913356	11514148 (96.65%)	150/144	709251 (7.94%)	163742 (1.37%)
S25	8953036	8872858 (99.10%)	150/144	37086 (0.55%)	77060 (0.86%)
S26	7907982	7837214 (99.11%)	150/146	29814 (0.5%)	67158 (0.85%)
S27	9268452	9185364 (99.10%)	150/146	29262 (0.42%)	80554 (0.87%)

Sample	Clean Reads Num	HQ Clean Reads Num(%)	Read Length	Adapter(%)	Low Quality(%)
S28	7625994	7554236 (99.06%)	150/146	35778 (0.62%)	68422 (0.9%)
S29	9014722	8930810 (99.07%)	150/146	40011 (0.6%)	79554 (0.88%)
S30	3102638	2830814 (91.24%)	150/144	391740 (16.84%)	26134 (0.84%)
S31	7710094	7549420 (97.92%)	150/145	215418 (3.72%)	78306 (1.02%)
S35	7550272	7475420 (99.01%)	150/143	21072 (0.37%)	73852 (0.98%)
S36	8702990	8623000 (99.08%)	150/143	26418 (0.4%)	77978 (0.9%)
S37	7680226	7516974 (97.87%)	150/142	212007 (3.68%)	86088 (1.12%)
S38	9560388	9469458 (99.05%)	150/142	26514 (0.36%)	89638 (0.94%)
S39	7470352	7402738 (99.09%)	150/142	26556 (0.48%)	65408 (0.88%)
S40	7893130	7821978 (99.10%)	150/142	24186 (0.41%)	69072 (0.88%)
S41	7020690	6954572 (99.06%)	150/142	19362 (0.36%)	65068 (0.93%)
S42	8668018	8585874 (99.05%)	150/144	30648 (0.47%)	80218 (0.93%)
S43	7353900	7289374 (99.12%)	150/144	28113 (0.5%)	62670 (0.85%)
S44	7444926	7376008 (99.07%)	150/144	27279 (0.48%)	67236 (0.9%)
S46	7315732	7250630 (99.11%)	150/144	32229 (0.58%)	62814 (0.86%)
S47	8268686	8195710 (99.12%)	150/146	39405 (0.64%)	69602 (0.84%)
S48	9144396	9062068 (99.10%)	150/146	34707 (0.51%)	79750 (0.87%)
S49	7999198	7925266 (99.08%)	150/146	35640 (0.6%)	70870 (0.89%)
S50	8443624	8365724 (99.08%)	150/146	40623 (0.64%)	74580 (0.88%)
S51	6868274	6728336 (97.96%)	150/145	154839 (3%)	60648 (0.88%)
S52	18429428	18264310 (99.10%)	150/145	82071 (0.59%)	160814 (0.87%)
S53	7525136	7345966 (97.62%)	150/145	240279 (4.26%)	75078 (1%)
S54	5718490	5615964 (98.21%)	150/143	105510 (2.46%)	49920 (0.87%)
S85	10871888	10773476 (99.09%)	150/143	39756 (0.48%)	96072 (0.88%)

Sample	Clean Reads Num	HQ Clean Reads Num(%)	Read Length	Adapter(%)	Low Quality(%)
S86	8163302	8081832 (99.00%)	150/143	32415 (0.53%)	79984 (0.98%)
S88	8690782	8499712 (97.80%)	150/142	261918 (4.02%)	98204 (1.13%)

Table S3 Statistics for raw data (RAD-seq) from the 49 *S. secundatum* accessions (cultivar)

Sample	HQ Clean Reads Number	Q30(%)	GC(%)	SNP count	Sample	HQ Clean Reads Number	Q30(%)	GC(%)	SNP count
S02	7461350	90.64%	44.03%	389390	S30	2830814	87.74%	43.57%	212295
S03	14191686	90.08%	44.78%	523574	S31	7549420	88.07%	44.03%	446466
S04	7875834	91.72%	42.07%	88359	S35	7475420	87.51%	43.10%	502711
S05	5871930	91.51%	42.43%	216097	S36	8623000	87.82%	43.11%	527045
S06	5391786	91.79%	42.64%	201504	S37	7516974	87.87%	43.90%	475685
S07	6710640	91.93%	42.73%	206981	S38	9469458	87.71%	43.41%	239624
S08	8816270	91.97%	42.41%	94858	S39	7402738	87.77%	43.27%	420225
S09	6772506	91.74%	42.27%	184687	S40	7821978	87.65%	43.07%	516710
S11	5705944	91.76%	42.33%	174940	S41	6954572	87.68%	43.20%	493311
S12	7625422	91.72%	42.38%	95270	S42	8585874	87.69%	43.30%	531081
S13	7422216	91.57%	42.26%	137431	S43	7289374	87.80%	43.21%	502312
S14	6994242	91.99%	42.23%	180814	S44	7376008	87.82%	43.18%	500653
S15	13574522	92.05%	42.47%	166716	S46	7250630	87.68%	43.60%	224254
S18	6579418	91.84%	42.50%	190834	S47	8195710	87.80%	43.48%	253164
S19	6902662	92.21%	42.27%	173850	S48	9062068	88.00%	43.45%	294178
S20	4470052	91.57%	43.37%	157793	S49	7925266	87.86%	43.61%	234335
S21	8661892	92.24%	42.86%	88299	S50	8365724	87.76%	43.55%	236261
S22	8897560	87.69%	43.25%	273956	S51	6728336	87.76%	43.67%	229236
S23	7785116	88.27%	44.26%	272710	S52	18264310	87.79%	43.53%	279854
S24	11514148	88.69%	45.35%	178015	S53	7345966	88.24%	44.12%	234572
S25	8872858	87.59%	43.43%	490513	S54	5615964	87.94%	43.67%	218805
S26	7837214	87.72%	43.26%	485131	S85	10773476	87.78%	43.54%	245564
S27	9185364	87.95%	43.24%	559090	S86	8081832	87.45%	43.51%	299202
S28	7554236	87.76%	43.37%	451149	S88	8499712	87.87%	44.20%	214921
S29	8930810	87.73%	43.38%	313743	Ave	8094088	89.16%	43.26%	298534

Table S4 Detailed information on polymorphism revealed by 28 SRAP primer pairs.

NO.	Primer combinations	TNB	NPL	PPL（%）	NO.	Primer combinations	TNB	NPL	PPL（%）
1	Em1-Me5	9	9	100%	15	Em10-Me13	9	9	100%
2	Em4-Me4	8	8	100%	16	Em10-Me14	10	10	100%
3	Em1-Me6	10	10	100%	17	Em11-Me10	9	9	100%
4	Em2-Me13	9	9	100%	18	Em11-Me16	8	8	100%
5	Em2-Me17	6	6	100%	19	Em12-Me1	10	10	100%
6	Em4-Me20	10	10	100%	20	Em14-Me4	6	6	100%
7	Em6-Me13	9	9	100%	21	Em14-Me8	8	7	87.50%
8	Em6-Me17	10	10	100%	22	Em15-Me2	7	7	100%
9	Em7-Me1	12	12	100%	23	Em15-Me8	13	13	100%
10	Em7-Me19	12	12	100%	24	Em17-Me2	12	11	91.70%
11	Em8-Me2	9	9	100%	25	Em18-Me10	12	12	100%
12	Em8-Me17	11	10	90.90%	26	Em18-Me16	10	10	100%
13	Em9-Me3	9	8	88.90%	27	Em20-Me4	12	12	100%
14	Em9-Me14	10	10	100%	28	Em20-Me11	13	12	92.30%

Note: TNB: Total number of bands; NPL: Number of polymorphic loci; PPL: Percentage of polymorphic loci.

Table S5 Genetic diversity parameters (h and I) of 46 *S. secundatum* accessions based on SRAP markers

Primer	*h*	*I*
Em1-Me5	0.4660	0.6587
Em4-Me4	0.4537	0.6461
Em1-Me6	0.4537	0.6461
Em2-Me13	0.4660	0.6587
Em2-Me17	0.4660	0.6587
Em4-Me20	0.4962	0.6894
Em6-Me13	0.4537	0.6461
Em6-Me17	0.4849	0.6779
Em7-Me1	0.4915	0.6846
Em7-Me19	0.4537	0.6461
Em8-Me2	0.4849	0.6779
Em8-Me17	0.4764	0.6693
Em9-Me3	0.4764	0.6693
Em9-Me14	0.4849	0.6779
Em10-Me13	0.4764	0.6693
Em10-Me14	0.4660	0.6587
Em11-Me10	0.4055	0.5954
Em11-Me16	0.4055	0.5954
Em12-Me1	0.4849	0.6779

Em14-Me4	0.4991	0.6922
Em14-Me8	0.4849	0.6779
Em15-Me2	0.4962	0.6894
Em15-Me8	0.4849	0.6779
Em17-Me2	0.4849	0.6779
Em18-Me10	0.4660	0.6587
Em18-Me16	0.4055	0.5954
Em20-Me4	0.4660	0.6587
Em20-Me11	0.4055	0.5954
**Mean**	**0.4657**	**0.6581**
**St. Dev**	**0.0283**	**0.0294**

Note: *h*: Nei's genetic diversity; *I*: Shannon's information index; St. Dev: Standard Deviation.

Table S7. Primer sequences and amplification results of ISSR analysis.

Primers	Primer sequences （5'–3'）	Annealing temperature /℃	Total number of bands （TNB）	Number of polymorphic bands（NPB）	Percentage of polymorpbic bands （PPB）
ISSR810	（GA）$_{8}$T	52	17	17	100%
ISSR811	（GA）$_{8}$C	55	14	14	100%
ISSR812	（GA）$_{8}$A	52	14	14	100%
ISSR815	（CT）$_{8}$G	55	22	22	100%
ISSR817	（CA）$_{8}$A	52	18	18	100%
ISSR820	（GT）$_{8}$C	55	20	20	100%
ISSR823	（TC）$_{8}$C	55	16	16	100%
ISSR825	（AC）$_{8}$T	52	18	18	100%
ISSR826	（AC）$_{8}$C	55	18	18	100%
ISSR827	（AC）$_{8}$G	55	21	21	100%
ISSR828	（TG）$_{8}$A	52	15	15	100%
ISSR834	（AG）$_{8}$YT	54	24	24	100%
ISSR835	（AG）$_{8}$YC	56	25	25	100%
ISSR836	（AG）$_{8}$YA	53	25	25	100%
ISSR843	（CT）$_{8}$RA	54	14	14	100%
ISSR845	（CT）$_{8}$RG	56	21	21	100%
ISSR846	（CA）$_{8}$RT	54	21	21	100%
ISSR847	（CA）$_{8}$RC	56	20	20	100%
ISSR848	（CA）$_{8}$RG	56	19	19	100%
ISSR855	（AC）$_{8}$YT	54	16	16	100%
ISSR856	（AC）$_{8}$YA	54	18	18	100%

ISSR880	（GGAGA）$_3$	53	18	18	100%
ISSR886	VDV（CT）$_7$	54	20	20	100%
ISSR889	DBD（AC）$_7$	53	23	23	100%
ISSR891	HVH（TG）$_7$	53	21	21	100%
ISSR899	CAT GGT GTT GGT CAT TGT TCC A	58	24	24	100%
ISSR900	ACT T（CC）$_2$A CAG GTT AA（CA ）$_2$	58	25	25	100%
Total			527	527	
Ave.			19.52	19.52	

Table S8 Genetic diversity parameters (h and I) of 46 *S. secundatum* accessions based on ISSR markers

Primer	*h*	*I*
ISSR810	0.4234	0.6145
ISSR811	0.4991	0.6922
ISSR812	0.4395	0.6314
ISSR815	0.4660	0.6587
ISSR817	0.4764	0.6693
ISSR820	0.4537	0.6461
ISSR823	0.4849	0.6779
ISSR825	0.4849	0.6779
ISSR826	0.4764	0.6693
ISSR827	0.4660	0.6587
ISSR828	0.4537	0.6461
ISSR834	0.4660	0.6587
ISSR835	0.3639	0.5501
ISSR836	0.4055	0.5954
ISSR843	0.4849	0.6779
ISSR845	0.4915	0.6846
ISSR846	0.4234	0.6145
ISSR847	0.3856	0.5740
ISSR848	0.4395	0.6314

ISSR855	0.4055	0.5954
ISSR856	0.4234	0.6145
ISSR880	0.4537	0.6461
ISSR886	0.4764	0.6693
ISSR889	0.4764	0.6693
ISSR891	0.4764	0.6693
ISSR899	0.4764	0.6693
ISSR900	0.4395	0.6314
**Mean**	**0.4523**	**0.6442**
**St. Dev**	**0.0343**	**0.0359**

Note: *h*: Nei's genetic diversity; *I*: Shannon's information index; St. Dev: Standard Deviation.

Table S10. Origins of the 49 *S. secundatum* accessions examined in this study.

No.	Accession No.	Specific (variety) Names	Origins
1	S02	*S. secundatum*	Hainan, China
2	S03	*S. secundatum*	Hainan, China
3	S04	*S. secundatum* var.*varietgatum*	Hainan, China
4	S05	*S. secundatum*	Guangxi, China
5	S06	*S. secundatum*	Guangdong, China
6	S07	*S. secundatum*	Guangdong, China
7	S08	*S. secundatum*	Guangxi, China
8	S09	*S. secundatum*	Guangxi, China
9	S11	*S. secundatum*	Hainan, China
10	S12	*S. secundatum* var.*varietgatum*	Hainan, China
11	S13	*S. secundatum*	Yunnan, China
12	S14	*S. secundatum*	Yunnan, China
13	S15	*S. secundatum*	Yunnan, China
14	S18	*S. secundatum*	Guangxi, China
15	S19	*S. secundatum*	Guangxi, China
16	S20	*S. secundatum* var.*varietgatum*	Hainan, China
17	S21	*S. secundatum* var.*varietgatum*	Hainan, China
18	S22	*S. secundatum*	Fujian, China
19	S23	*S. secundatum*	Fujian, China
20	S24	*S. secundatum*	Hainan, China
21	S25	*S. secundatum*	Fujian, China
22	S26	*S. secundatum*	Guangdong, China

23	S27	*S. secundatum*	Yunnan, China
24	S28	*S. secundatum*	Hainan, China
25	S29	*S. secundatum*	Hainan, China
26	S30	*S. secundatum*	Hainan, China
27	S31	*S. secundatum*	Yunnan, China
28	S35	*S. secundatum*	Guangdong, China
29	S36	*S. secundatum*	Hainan, China
30	S37	*S. secundatum*	Guangxi, China
31	S38	*S. secundatum* var.*varietgatum*	Guangxi, China
32	S39	*S. secundatum*	Hainan, China
33	S40	*S. secundatum*	Guangxi, China
34	S41	*S. secundatum*	Guangxi, China
35	S42	*S. secundatum*	Guangxi, China
36	S43	*S. secundatum*	Guangxi, China
37	S44	*S. secundatum*	Guangxi, China
38	S46	*S. secundatum*	Guangdong, China
39	S47	*S. secundatum*	Jiangsu, China
40	S48	*S. secundatum* var.*varietgatum*	Hainan, China
41	S49	*S. secundatum*	Fujian, China
42	S50	*S. secundatum*	Fujian, China
43	S51	*S. secundatum*	Fujian, China
44	S52	*S. secundatum*	Fujian, China
45	S53	*S. secundatum*	Fujian, China
46	S54	*S. secundatum 'jiajian II'*	Fujian, China
47	S85	*S. secundatum*	Hainan, China

48	S86	*S. secundatum*	Guangdong, China
49	S88	*S. secundatum*	Vanuatu, South Pacific

Table S11. Primer sequences used for SRAP analysis of *S. secundatum*.

Code	Forward Primers(5'-3')	Reverse Primers(5'-3')
Em1-Me5	TGAGTCCAAACCGGATA	GACTGCGTACGAATTAAT
Em4-Me4	TGAGTCCAAACCGGAAG	GACTGCGTACGAATTACG
Em1-Me6	TGAGTCCAAACCGGACA	TGAGTCCAAACCGGATA
Em2-Me13	TGAGTCCAAACCGGTAG	GACTGCGTACGAATTAAC
Em2-Me17	TGAGTCCAAACCGGTGC	GACTGCGTACGAATTAAC
Em4-Me20	TGAGTCCAAACCGGGAC	GACTGCGTACGAATTACG
Em6-Me13	TGAGTCCAAACCGGTAG	GACTGCGTACGAATTTAG
Em6-Me17	TGAGTCCAAACCGGTGC	GACTGCGTACGAATTTAG
Em7-Me1	TGAGTCCAAACCGGAAA	GACTGCGTACGAATTTGA
Em7-Me19	TGAGTCCAAACCGGCTA	GACTGCGTACGAATTTGA
Em8-Me2	TGAGTCCAAACCGGAAT	GACTGCGTACGAATTTGC
Em8-Me17	TGAGTCCAAACCGGTGC	GACTGCGTACGAATTTGC
Em9-Me3	TGAGTCCAAACCGGAAC	GACTGCGTACGAATTTCA
Em9-Me14	TGAGTCCAAACCGGTTG	GACTGCGTACGAATTTCA
Em10-Me13	TGAGTCCAAACCGGTAG	GACTGCGTACGAATTTCG
Em10-Me14	TGAGTCCAAACCGGTTG	GACTGCGTACGAATTTCG
Em11-Me10	TGAGTCCAAACCGGAGA	GACTGCGTACGAATTCAA
Em11-Me16	TGAGTCCAAACCGGTGT	GACTGCGTACGAATTCAA

Em12-Me1	TGAGTCCAAACCGGAAA	GACTGCGTACGAATTCAT
Em14-Me4	TGAGTCCAAACCGGAAG	GACTGCGTACGAATTCAG
Em14-Me8	TGAGTCCAAACCGGACC	GACTGCGTACGAATTCAG
Em15-Me2	TGAGTCCAAACCGGAAT	GACTGCGTACGAATTCTA
Em15-Me8	TGAGTCCAAACCGGACC	GACTGCGTACGAATTCTA
Em17-Me2	TGAGTCCAAACCGGAAT	GACTGCGTACGAATTCTC
Em18-Me10	TGAGTCCAAACCGGAGA	GACTGCGTACGAATTCTG
Em18-Me16	TGAGTCCAAACCGGTGT	GACTGCGTACGAATTCTG
Em20-Me4	TGAGTCCAAACCGGAAG	GACTGCGTACGAATTCGA
Em20-Me11	TGAGTCCAAACCGGAGC	GACTGCGTACGAATTCGA

Table S12. Primer sequences used for ISSR analysis of *S. secundatum*.

Code	Primer sequence (5'-3')	Code	Primer sequence (5'-3')
ISSR810	GAG AGA GAG AGA GAG AT	ISSR843	CTC TCT CTC TCT CTC TRA
ISSR811	GAG AGA GAG AGA GAG AC	ISSR845	CTC TCT CTC TCT CTC TRG
ISSR812	GAG AGA GAG AGA GAG AA	ISSR846	CAC ACA CAC ACA CAC ART
ISSR815	CTC TCT CTC TCT CTC TG	ISSR847	CAC ACA CAC ACA CAC ARC
ISSR817	CAC ACA CAC ACA CAC AA	ISSR848	CAC ACA CAC ACA CAC ARG
ISSR820	GTG TGT GTG TGT GTG TC	ISSR855	ACA CAC ACA CAC ACA CYT
ISSR823	TCT CTC TCT CTC TCT CC	ISSR856	ACA CAC ACA CAC ACA CYA
ISSR825	ACA CAC ACA CAC ACA CT	ISSR880	GGA GAG GAG AGG AGA
ISSR826	ACA CAC ACA CAC ACA CC	ISSR886	VDV CTC TCT CTC TCT CT
ISSR827	ACA CAC ACA CAC ACA CG	ISSR889	DBD ACA CAC ACA CAC AC
ISSR828	TGT GTG TGT GTG TGT GA	ISSR891	HVH TGT GTG TGT GTG TG
ISSR834	AGA GAG AGA GAG AGA GYT	ISSR899	CAT GGT GTT GGT CAT TGT TCC A
ISSR835	AGA GAG AGA GAG AGA GYC	ISSR900	ACT TCC CCA CAG GTT AAC ACA
ISSR836	AGA GAG AGA GAG AGA GYA		
