# Supplementary material for: Phenotypic and molecular marker analysis uncovers the genetic diversity of the grass Stenotaphrum secundatum
Source: BMC Genet. 2020 Aug 12;21:86. doi: 10.1186/s12863-020-00892-w (PMC7425169; doi:10.1186/s12863-020-00892-w)
Supplement: Supplementary file 2 — Additional file 2: Figure S1. Principal component analysis of 49 S. secundatum accessions. The contribution ratios of the first principal component and the second principal component were 56.23 and 15.87%, respectively. Note: The codes correspond to those listed in Additional file 1: Table S10. Figure S2. Statistics of SNP types in 49 S. secundatum accessions. Figure S3. PCA of 49 S. secundatum accessions (cultivars) based on SNP data. Figure S4. Sample amplification profiles of 46 S. secundatum germplasms based on SRAP primers EM14-ME4. M: 100 bp marker. Note: The codes correspond to those listed in Additional file 1: Table S10. Figure S5. Sample amplification profiles of 46 S. secundatum germplasms based on ISSR primer ISSR889. M: 100 bp marker. Note: The codes correspond to those listed in Additional file 1: Table S10. [file 12863_2020_892_MOESM2_ESM.pdf]

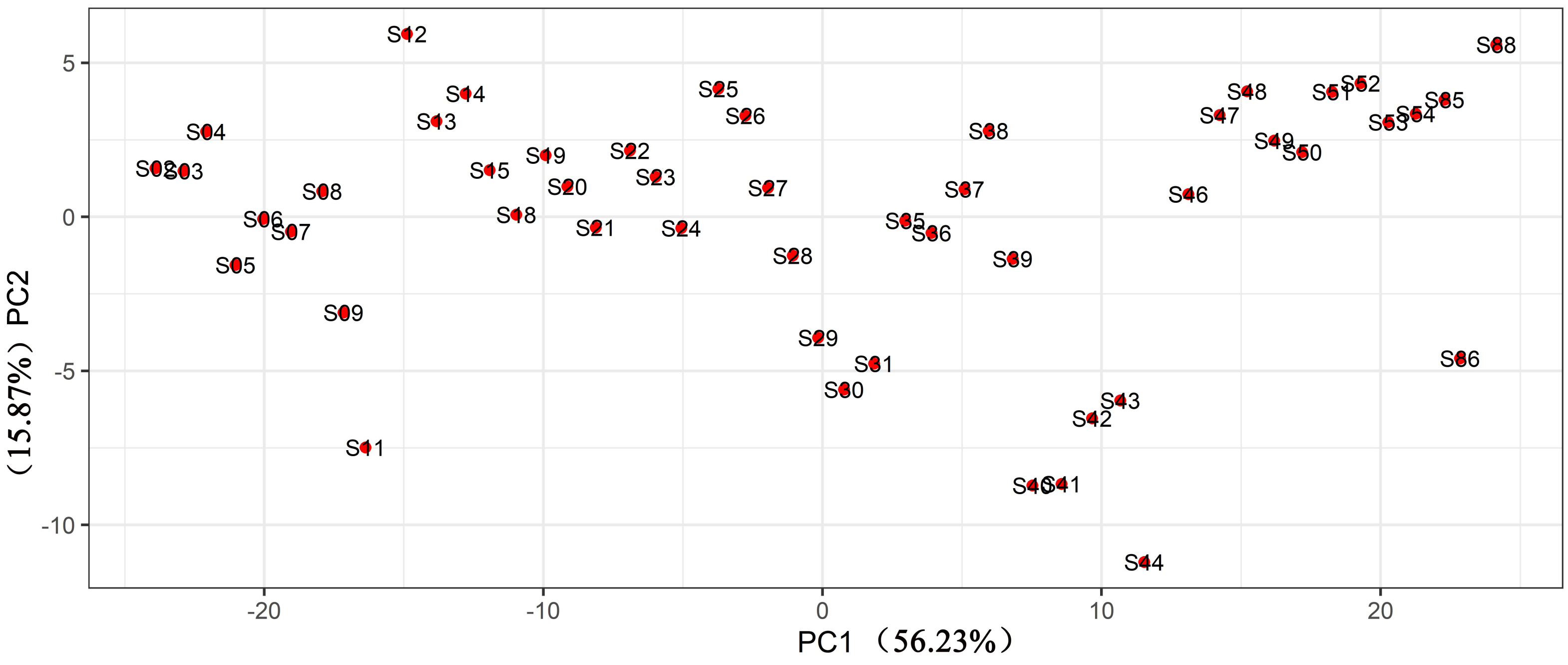

Figure S1 Principal component analysis of 49 *S. secundatum* accessions.

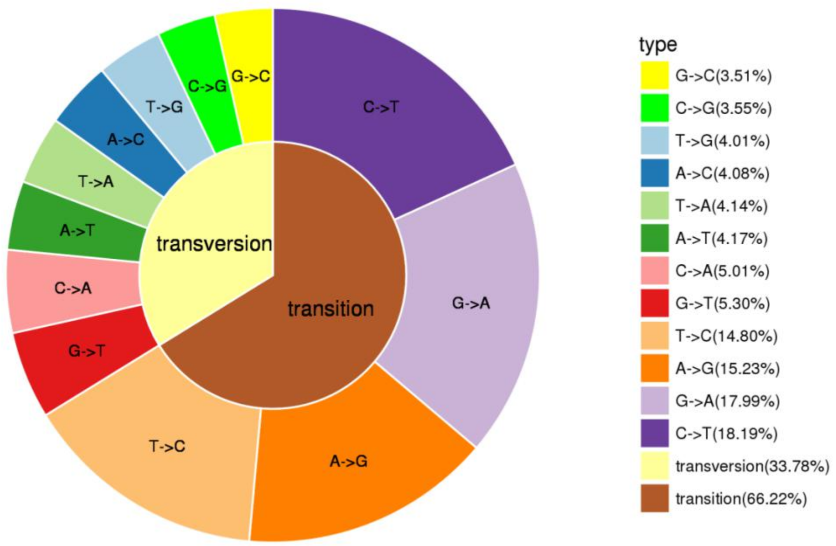

Figure S2 Statistics of SNP types in 49 *S. secundatum* accessions.

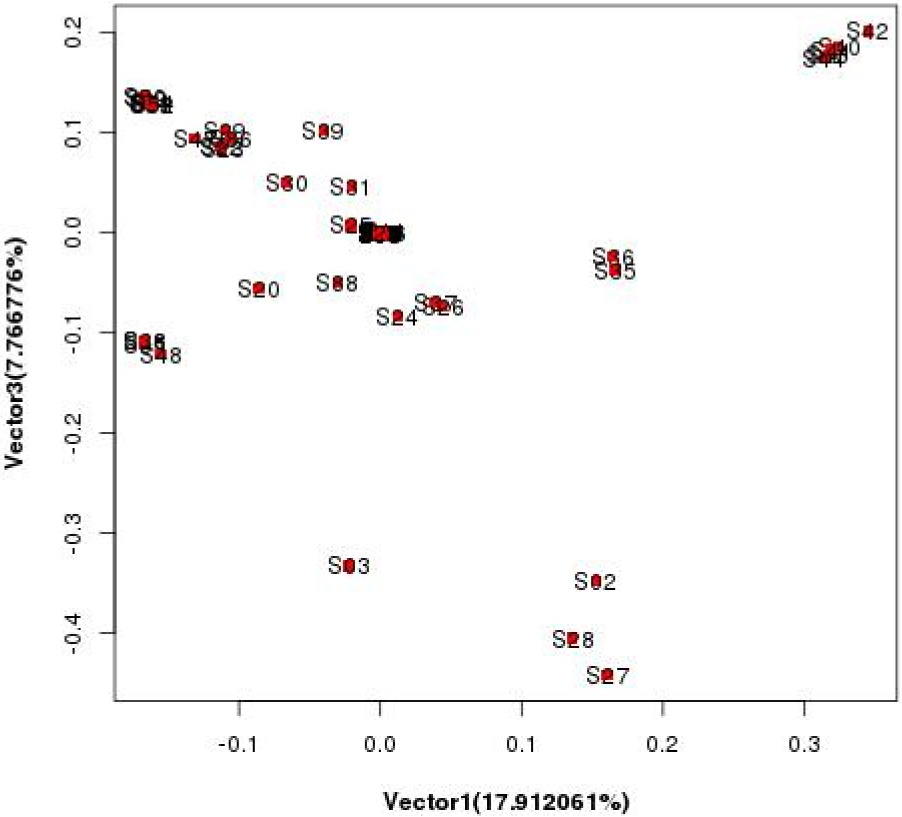

Figure S3 PCA of 49 *S. secundatum* accessions (cultivars) based on SNP data.

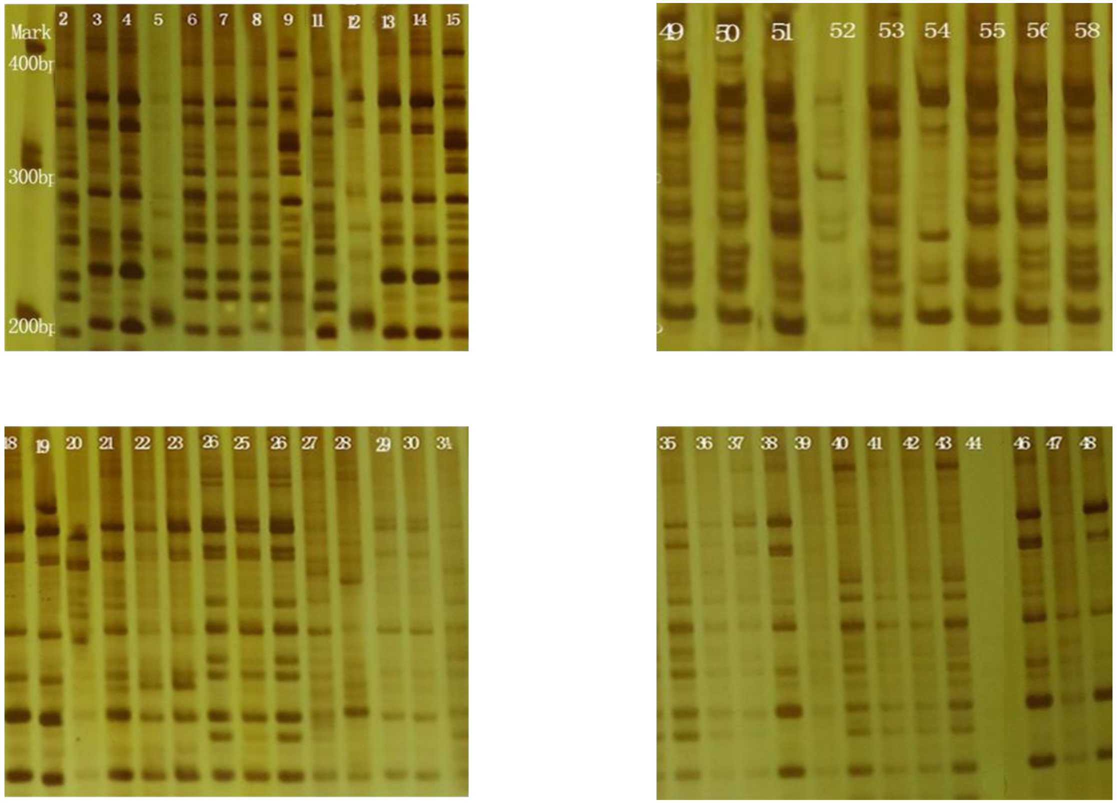

Figure S4 Sample amplification profiles of 46 *S. secundatum* germplasms based on SRAP primers EM14-ME4. M: 100 bp marker. Note: The codes correspond to those listed in Additional file 1: Table S10.

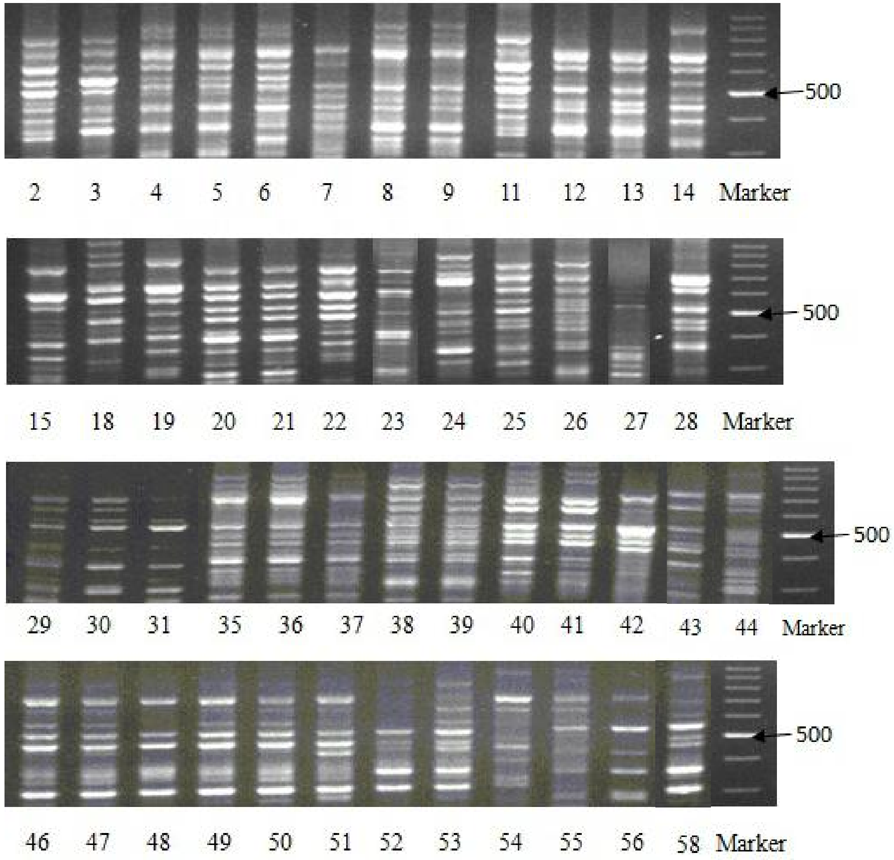

Figure S5 Sample amplification profiles of 46 *S. secundatum* germplasms based on ISSR primer ISSR889. M: 100 bp marker. Note: The codes correspond to those listed in Additional file 1: Table S10.
